# Perceived Discrimination and Health among Immigrants in Europe According to National Integration Policies

**DOI:** 10.3390/ijerph120910687

**Published:** 2015-08-31

**Authors:** Carme Borrell, Laia Palència, Xavier Bartoll, Umar Ikram, Davide Malmusi

**Affiliations:** 1 Agència de Salut Pública de Barcelona, Barcelona 08023, Spain; E-Mails: lpalenci@aspb.cat (L.P.); xbartoll@aspb.cat (X.B.); dmalmusi@aspb.cat (D.M.); 2 Ciber de Epidemiología y Salud Pública, 28029, Spain; 3 Universitat Pompeu Fabra, Barcelona 08003, Spain; 4 Institut de Recerca Biomèdica Sant Pau (IIB Sant Pau), Barcelona 08025, Spain; 5 Academic Medical Center, University of Amsterdam, Amsterdam 1100 DD, the Netherlands; E-Mail: u.ikram@amc.uva.nl

**Keywords:** discrimination, immigrant generation, national immigrant integration policy, low income countries, perceived health, depression

## Abstract

*Background*: Discrimination harms immigrants’ health. The objective of this study was to analyze the association between perceived discrimination and health outcomes among first and second generation immigrants from low-income countries living in Europe, while accounting for sex and the national policy on immigration. *Methods*: Cross-sectional study including immigrants from low-income countries aged ≥15 years in 18 European countries (European Social Survey, 2012) (sample of 1271 men and 1335 women). The dependent variables were self-reported health, symptoms of depression, and limitation of activity. The independent variables were perceived group discrimination, immigrant background and national immigrant integration policy. We tested for association between perceived group discrimination and health outcomes by fitting robust Poisson regression models. *Results*: We only observed significant associations between perceived group discrimination and health outcomes in first generation immigrants. For example, depression was associated with discrimination among both men and women (Prevalence Ratio-, 1.55 (95% CI: 1.16–2.07) and 1.47 (95% CI: 1.15–1.89) in the multivariate model, respectively), and mainly in countries with assimilationist immigrant integration policies. *Conclusion*: Perceived group discrimination is associated with poor health outcomes in first generation immigrants from low-income countries who live in European countries, but not among their descendants. These associations are more important in assimilationist countries.

## 1. Introduction

Discrimination has been described as “a socially structured and sanctioned phenomenon, justified by ideology and expressed in interactions between individuals and institutions, that preserves privileges for dominant groups at the cost of deprivation for others” [1]. There are many types of discrimination due to age, disability, gender, nationality, race, ethnicity, religion, and others, and immigrants usually suffer one or more of these types of discrimination [2]. Several studies and reviews, mainly focused on the USA, have shown that discrimination harms immigrants’ health [3,4,5,6,7,8]. Krieger recently reviewed this topic and identified 40 review articles on racial discrimination and health, representing over 350 original articles [1]. She found that most studies focused on interpersonal discrimination, and that there is a paucity of research on structural discrimination as a determinant of health inequalities.

In 2013, there were 33.5 million people living in the EU-27 member states that had been born outside the EU-27, and 17.3 million who had been born in a different EU-27 Member State [9], representing an historically high level of immigration [10]. Previous studies in Europe have found that immigrants from countries with a lower level of socioeconomic development, those with non-Christian faith, who speak a minority language at home, or who are unemployed are more likely to suffer discrimination [11]. Moreover, second generations do not perceive less discrimination than first generation immigrants, contrary to the authors’ hypothesis. However, not all countries have the same levels of discrimination against immigrants [12].

European countries have different policies for integrating migrant populations, in terms of family reunion, education, political participation, long-term residence, access to nationality, anti-discrimination and labor market mobility. Meuleman [10] classified European countries into three policy models based on how they rank in each of these dimensions, according to the Migrant Integration Policy Index (MIPEX): the inclusive model has inclusive policies in all dimensions; the assimilationist model offers relatively easy access to nationality but limited access to the labor market and family reunion, and puts little emphasis on anti-discrimination policies; and the exclusionist model excludes immigrants from most spheres of life and perceives immigrants as “temporary guests”. These policy models may shape the context in which immigrants experience discrimination, and how this affects their health. However, there are no studies that analyze discrimination and health among first and second generation immigrants in European countries and that consider whether the effects of discrimination on health outcomes change in countries with different integration policy.

The hypothesis of this research was that perceived discrimination changes according to immigrants’ generation, sex and national integration policy model. Perceived discrimination would be associated with poor health outcomes, and these associations would change between immigrant generations, with the first generation immigrants having less opportunities to be integrated in the destination country. Moreover, we hypothesized differences depending on the type of integration policy of the destination country, with inclusive countries having a lower prevalence of perceived discrimination and weaker associations with health outcomes. Finally, since women have poorer health status and mental health than men, we were interested in testing whether these associations differed between genders, as has been shown in previous studies [13].

Therefore, the objective of this study was to analyze the association between perceived discrimination and health outcomes among first and second generation immigrants from low-income countries living in Europe, while accounting for sex and national policy on immigration.

## 2. Methods

### 2.1. Design, Study Population, Sample and Information Sources

We performed a cross-sectional study of residents aged ≥15 years in 18 European countries who were first or second generation immigrants, namely those born abroad in a low-income country, or whose parents were born in a low-income country, respectively. We broadly defined low-income countries as those not included in the International Monetary Fund list of advanced economies [14]. In this selection, we excluded individuals who migrated to the same country before 1992 (e.g., from Russia to Lithuania).

Data were obtained from the 6th round of the European Social Survey (2012), a cross-national survey that uses representative samples of all persons aged ≥15 years resident in private households in European countries [15]. Participants were selected by strict random probability methods at every stage. Substitution of non-responding households or individuals was not permitted, regardless of whether they were “refusals”, “non-contacts” or “ineligibles”. Response rates ranged from 33.8% in Germany to 77.1% in Portugal [16]. Residents in Hungary, the Czech Republic, Bulgaria, Poland and Slovakia were excluded due to the small number of immigrants (<25 cases). The study sample included 1271 men and 1335 women.

### 2.2. Variables

#### 2.2.1. Dependent Variables

Self-reported health status was evaluated using the question, “How is your health in general?” (“very good”, “good”, “fair”, “bad”, “very bad”). Answers were dichotomized to create the variable *poor health* (yes/no), with “yes” including the categories fair, bad and very bad [17].

Symptoms of depression were evaluated using the self-reported Depression Scale, CES-D (short version) from the Center for Epidemiologic Studies. This scale includes 8 items, beginning with the question, “How often you have felt this way during the past week?” depressed; that everything you did was an effort; that your sleep was restless; happy; lonely; enjoyed life; sad; and could not get going. Using a 4-point scale for each item (0–3; negative items: 0 = never or almost never, 3 = all or almost all of the time; *vice versa* for positive items) we calculated a sum score ranging from 0 to 24. Since several cut-off points have been used in previous studies [18,19], we established >8 as the cut-off point for depression, which included a 25% of the sample.

Limitation of activity was evaluated using the question, “Are you hampered in any way in your daily activities by any longstanding illness or disability, ailment or any health problem?” (“yes, a lot”, “yes, to some extent”, “no”). Individuals who answered “yes, a lot” or “yes, to some extent” were considered to have a limitation.

#### 2.2.2. Independent Variables

Perceived group discrimination, evaluated using the following two questions: “Would you describe yourself as belonging to a group that is discriminated against in this country?” (yes/no); and “On what grounds is your group discriminated against?” (color or race, nationality, religion, language, ethnic group, age, gender, sexuality, disability, other, each answered as yes/no). Individuals who answered “yes” to the first question and “colour/race, nationality, religion, language or ethnic group” to the second question were considered to perceive group discrimination.

Immigrant background:

First generation: born in a low income country.

Second generation: both mother and father born in a low income country.

National immigrant integration policy: MIPEX uses 144 policy indicators developed to create a rich, multi-dimensional picture of migrants’ opportunities for societal participation. Countries were placed in 3 groups according to Meuleman’s classification [10]:

Inclusive countries: Belgium, Spain, Finland, UK, Italy, Netherlands, Norway, Portugal, Sweden.

Assimilationist countries: Switzerland, Germany, France, Ireland

Exclusionist countries: Cyprus, Denmark, Estonia, Lithuania, Slovenia.

#### 2.2.3. Other Variables

Age, sex, citizenship, educational level (primary or less, lower secondary, upper secondary and tertiary), marital status (married/cohabiting, separated/divorced/widowed, never married), activity (paid work, studying, unemployed, retired or disabled, housework, others).

### 2.3. Data Analysis

**Figure 1:**
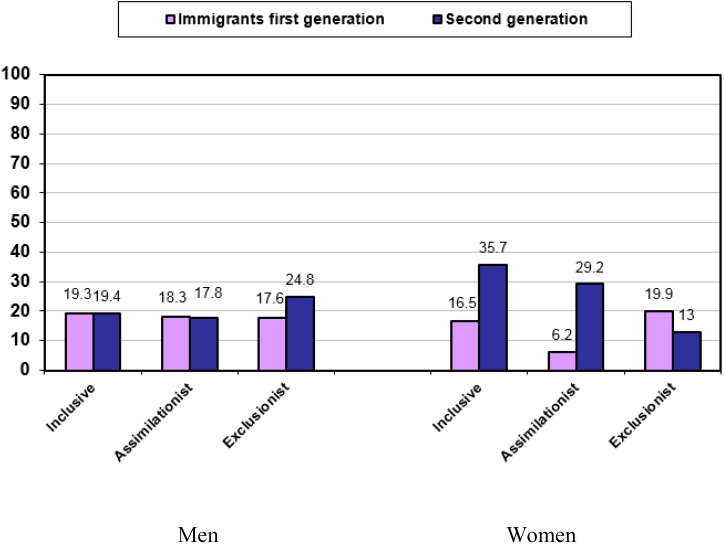
Prevalence of perceived group discrimination among first and second generation immigrants, according to sex and national immigrant integration policy.

All analyses were stratified by sex. We performed a descriptive analysis of all variables for each generation of immigrant, and type of national integration policy (Table 1 and Figure 1).

To analyze the association between perceived group discrimination and health outcomes, we computed the prevalence of poor health outcomes among those who felt discriminated against and those who did not and compared them through Chi-square test. Moreover, we fitted robust Poisson regression models to obtain prevalence ratios between these groups [20] (two adjustment models: (a) adjusted by age; (b) adjusted by age, citizenship, educational level, marital status and activity). We conducted separate analyses for each generation (Table 2), and since we only found statistically significant associations in first generation immigrants, we only tested for association between perceived group discrimination and health outcomes for each immigrant integration policy in first generation immigrants (Table 3).

All calculations were weighted by combining two weights. The first weight accounts for differences in the probability of inclusion, and thus corrects for bias in the sampling design, and uses auxiliary information to reduce sampling error and potential non-response bias. The second weight accounts for population size, and corrects for the fact that most countries in the European Social Survey have different population sizes but similar sample sizes. Weights were additionally divided by their mean for all countries in each type of national integration policy, so that actual sample sizes for each type of national country integration policy were maintained (otherwise, groups of countries with small populations would be poorly represented). All analyses were performed using SPSS.

**Table 1:** Sample descriptive percentages, according to immigrant background, sex and national immigrant integration policy.

Socio-demographic variables	1st Generation						2nd Generation					
	Men			Women			Men			Women		
	Inclusive (n = 536)	Assimilationist (n = 319)	Exclusionist (n = 105)	Inclusive (n = 524)	Assimilationist (n = 326)	Exclusionist (n = 154)	Inclusive (n = 72)	Assimilationist (n = 90)	Exclusionist (n = 102)	Inclusive (n = 100)	Assimilationist (n = 96)	Exclusionist (n = 80)
***Citizenship (%)***												
**Yes**	52.4	66.6	51.5	55.6	69.4	41.5	98.5	80.9	76.0	99.5	84.2	80.5
**No**	47.6	33.4	48.5	43.5	30.6	58.5	1.5	19.1	24.0	0.5	15.8	19.5
**Missing**	−	−	−	0.9	−	−	−	−	−	−	−	
***Educational level (%)***												
**Primary or less **	18.1	14.0	6.0	22.3	13.8	4.6	3.3	13.5	2.5	7.7	7.8	5.3
**Lower Secondary**	31.8	43.4	29.4	31.1	40.5	29.5	33.0	51.5	33.3	37.4	68.2	27.8
**Upper Secondary**	19.9	19.5	29.9	22.3	18.8	36.8	36.8	22.4	45.7	24.6	18.2	43.5
**Tertiary**	22.4	23.0	34.5	19.7	26.7	28.3	26.1	12.6	18.5	30.3	5.6	23.3
**Missing**	7.7	0.1	0.3	4.6	0.2	0.9	0.8	−	−	−	0.1	−
***Marital status (%)***												
**Married/cohabiting**	57.8	68.9	62.6	57.3	59.7	61.0	29.2	33.2	39.3	30.2	26.8	44.4
**Separated/divorced/widowed**	4.9	7.6	10.7	16.2	18.2	19.1	2.9	3.8	8.7	3.9	13.4	17.2
**Never married**	35.5	23.4	26.3	24.0	22.0	19.0	66.5	63.0	49.8	65.9	59.8	36.6
**Missing**	1.8	0.1	0.3	2.5	0.1	1.0	1.4	−	2.2	−	−	1.8
***Activity (%)***												
**Paid work**	57.0	57.8	64.7	44.4	41.5	38.5	47.3	45.7	57.0	51.9	23.1	36.7
**Studying**	10.0	7.7	7.3	6.7	5.5	12.8	32.5	31.9	23.2	24.3	39.0	27.5
**Unemployed**	17.6	12.0	12.6	12.9	10.4	17.1	16.1	10.3	7.9	11.5	11.0	9.2
**Retired/disabled**	10.6	19.6	3.4	12.6	16.4	4.7	1.6	10.5	8.8	2.8	13.1	15.2
**Housework**	1.4	0.7	1.1	21.0	24.3	22.4	−	−	−	8.8	13.9	8.9
**Other **	3.1	1.5	2.5	0.9	1.4	−	2.5	1.5	−	0.7	−	2.4
**Missing**	0.2	0.7	8.4	1.4	0.4	4.4	−	−	3.2	−	−	−
**Age (median)**	38.0	42.4	45.0	42.0	44.0	39.0	25.8	26.5	33.4	30.0	22.0	36.0

Note: n = number of cases.

**Table 2:** Prevalence of health outcomes. Association of perceived group discrimination with poor health outcomes (prevalence ratios): age adjusted model and all adjusted; men and women by immigration background.

Health Outcomes, Sex and Discrimination	1st Generation			2nd Generation		
	Prev	PR_age_ (95% CI)	PR_full_ (95% CI)	Prev	PR_age_ (95% CI)	PR_full_ (95% CI)
Poor self-perceived health						
Men						
Not discriminated	26.8	1	1	31.3	1	1
Discriminated	24.7	0.90 (0.66–1.21)	0.94 (0.71–1.25)	23.6	0.66 (0.35–1.24)	0.71 (0.40–1.28)
Women						
Not discriminated	33.1	1	1	31.3	1	1
Discriminated	36.1	1.05 (0.81–1.36)	1.31 (1.04–1.66) *	19.2	0.76 (0.46–1.26)	0.77 (0.43–1.38)
				*		
Depression						
Men						
Not discriminated	18.8	1	1	18.9	1	1
Discriminated	29.7	1.59 (1.19–2.12) **	1.55 (1.16–2.07) **	20.8	1.06 (0.53–2.13)	0.78 (0.42–1.45)
	**					
Women						
Not discriminated	26.2	1	1	24.1	1	1
Discriminated	44.2	1.66 (1.30–2.13) ***	1.47 (1.15–1.89) **	17.8	0.58 (0.30–1.13)	0.61 (0.30–1.22)
	***					
Limitation of activity						
Men						
Not discriminated	19.0	1	1	20.3	1	1
Discriminated	24.7	1.50 (1.13–1.99) **	1.49 (1.13–1.98) **	18.2	1.15 (0.62–2.14)	1.01 (0.57–1.79)
Women						
Not discriminated	17.9	1	1	18.7	1	1
Discriminated	27.1	1.55 (1.08–2.23) *	1.51 (1.08–2.11) *	19.2	1.16 (0.66–2.05)	1.30 (0.77–2.19)
	*					

Notes: * *p* ≤ 0.05, ** *p* ≤ 0.01, *** *p* ≤ 0.001 (*p* value of comparing Prevalence among discriminated and not discriminated and *p* value of PR); PR, Prevalence Ratio; 95%CI, 95% confidence interval; full, model adjusted for age, citizenship, educational level, marital status, activity.

**Table 3:** Prevalence of health outcomes. Association of perceived group discrimination with poor health outcomes (prevalence ratios): age adjusted model and all adjusted; by country integration policy; men and women immigrants of first generation.

Health Outcomes, Sex and Discrimination	Inclusive			Assimilationist			Exclusionist		
	Prev	PR_age_ (95% CI)	PR_full_ (95% CI)	Prev	PR_age_ (95% CI)	PR_full_ (95% CI)	Prev	PR_age_ (95% CI)	PR_full_ (95% CI)
**Poor self-perceived health**									
**Men**									
** Not discriminated**	23.7	1	1	31.4	1	1	28.6	1	1
** Discriminated**	25.5	0.97 (0.66–1.42)	0.84 (0.56–1.25)	31.0	1.01 (0.66–1.55)	1.05 (0.66–1.66)	5.3	0.38 (0.05–2.64)	0.25 (0.03–1.76)
							*		
**Women**									
** Not discriminated**	32.2	1	1	37.4	1	1	25.6	1	1
** Discriminated**	22.6	0.59 (0.36–0.96) *	0.79 (0.51–1.21)	60.0	1.92 (1.40–2.63) ***	1.93 (1.34–2.78) ***	58.6	2.51 (1.72–3.66) ***	2.79 (1.54–5.05) **
				*			**		
**Depression**									
**Men**									
** Not discriminated**	20.8	1	1	16.4	1	1	15.7	1	1
** Discriminated**	30.3	1.40 (0.96–2.03)	1.48 (1.02–2.13) *	30.4	2.21 (1.32–3.68) *	2.23 (1.27–3.92) **	23.5	1.41 (0.53–3.76)	2.10 (0.67–6.61)
	*			*					
**Women**									
** Not discriminated**	26.9	1	1	27.4	1	1	20.6	1	1
** Discriminated**	47.6	1.72 (1.26–2.36) **	1.40 (1.00–1.97) *	52.6	1.88 (1.17–3.01) **	1.87 (1.23–2.84) **	28.6	1.55 (0.72–3.37)	0.25 (0.08–0.79) *
	***			*					
**Limitation of activity**									
**Men**									
** Not discriminated**	14.5	1	1	24.7	1	1	23.8	1	1
** Discriminated**	16.5	0.95 (0.59–1.53)	1.14 (0.72–1.79)	43.1	2.30 (1.60–3.30) ***	2.07 (1.41–3.05) ***	11.1	0.82 (0.21–3.28)	0.86 (0.24–3.08)
				**					
**Women**									
** Not discriminated**	11.5	1	1	25.9	1	1	20.5	1	1
** Discriminated**	20.2	2.38 (1.47–3.87) ***	2.37 (1.30–4.31) **	35.0	1.54 (0.88–2.69)	1.73 (1.01–2.96) *	41.4	1.19 (0.54–2.62)	1.86 (0.91–3.81)
	*						*		

Notes: * *p* ≤ 0.05, ** *p* ≤ 0.01, *** *p* ≤ 0.001 (*p* value of comparing Prevalence among discriminated and not discriminated and *p* value of PR); PR, Prevalence Ratio; 95% CI, 95% confidence interval; full, model adjusted for age, citizenship, educational level, marital status, activity.

## 3. Results

The results of the descriptive analysis for each generation of immigrants, sex and type of national immigration policy are shown in Table 1. Most immigrants were first generation immigrants. Among first generation immigrants, attainment of citizenship was highest in assimilationist countries, reaching almost 70% in this group. Most first generation immigrants had secondary or lower educational level, were married or cohabitating, and were in paid work (men) or paid work or housework (women). The median age was approximately 40 years. In contrast, second generation of immigrants had a notably different profile: they were generally younger (20–30 years), almost all had citizenship of the country of residence, and most were never married, and were in paid work or studying.

In women, perceived group discrimination was most common among second generation immigrants living in inclusive (35.7%) or assimilationist (29.2%) countries (Figure 1). Among men, the highest percentage was observed among second generation immigrants in exclusionist countries (24.8%).

The results of the analysis of association between perceived discrimination and health outcomes in first and second generation immigrants are shown in Table 2. The prevalence of poor health outcomes was higher among women than men. Among individuals who perceived group discrimination, the prevalence of poor health outcomes was higher in first than in second generation immigrants. We only observed significant associations between perceived group discrimination and health outcomes among first generation immigrants: poor self-perceived health in women (PR 1.31, 95% CI 1.04–1.66 in the full multivariate model); depression in both men (PR 1.55, 95% CI 1.16–2.07 in the full multivariate model) and women (PR 1.47, 95% CI 1.15–1.89 in the full multivariate model); and limitation of activity in men (PR 1.49, 95% CI 1.13–1.98 in the full multivariate model) and women (PR 1.51, 95% CI 1.08–2.11 in the full multivariate model).

The results of the analysis of association between perceived discrimination and health outcomes in first generation immigrants for each sex and type of national integration policy are shown in Table 3. In inclusive countries, the multivariate models show a positive association between perceived discrimination and depression, among both men and women, and limitation of activity among women. In assimilationist countries, perceived discrimination was associated with all health outcomes except poor self-perceived health among men. For example, in the multivariate model the PR for depression was 2.23 in men (95% CI 1.27–3.92) and 1.87 in women (95% CI 1.23–2.84). In exclusionist countries, perceived discrimination was associated with poor self-perceived health among women.

## 4. Discussion

In this study we show that: (a) the prevalence of perceived group discrimination varies according to sex, immigrant’s generation and type of national integration policy models; (b) perceived group discrimination was associated with poor health outcomes, but only in first generation immigrants; (c) perceived group discrimination was more consistently associated with health outcomes in assimilationist countries than in other groups of countries.

The prevalence of perceived group discrimination was not higher among first generation than second generation immigrants. André *et al.* [12] reported perceived group discrimination in persons with an immigrant background, based on data from the European Social Survey (2004–2007), and did not find differences between first and second generations. These authors found that perceived group discrimination depended less on the destination country and more on the country of origin and the individual characteristics of the immigrant (perceived group discrimination was lower among individuals with citizenship or who had a native parent, and higher among those who speak a minority language at home). Integration policies based on long-term residence and family reunion were associated with lower levels of perceived group discrimination. Stratifying by sex and national policy model, we observed differences between generations in the prevalence of perceived discrimination among women, with greater perceived discrimination among second generation immigrants in inclusive and assimilationist countries, but not in exclusionist countries. Note that the median age of second generation immigrant women was higher in exclusionist countries than in other countries. Among men, we observed no notable generational differences in the prevalence of perceived group discrimination in inclusive and assimilationist countries, but a higher prevalence among second generation immigrant men in exclusionist countries.

The prevalence of poor health outcomes was higher among first generation immigrants, as previously observed by Missinne and Bracke [21] and Leveque *et al.* [22] using the third wave of European Social Survey (2006). Levecque *et al.* [22] suggest that this higher prevalence is partly due to perceived discrimination and barriers to socioeconomic integration. In fact, we did not observe any differences in health status between first and second generations who did not feel discriminated against, but rather only among immigrants who perceived discrimination. We only observed associations between perceived group discrimination and health outcomes in first generation immigrants, mainly for depression. Altered mental health is one of the most widely recognized health outcomes associated with discrimination in general [1], and particularly among immigrants, such as in Asian Americans in the U.S. [4,5]. To our knowledge, these associations have not previously been examined Europe-wide, and our results suggest that perceived group discrimination has a negative impact on the health of first generation immigrants, who probably are less well-integrated into the destination country. It is worth mentioning that in this study perceived discrimination was measured at group and not individual level, so while second generations may feel that they belong to a discriminated group, they may not experience discrimination themselves; this is referred to as the “discrepancy between individual and group discrimination” [1].

However, US studies have found that the duration of residence in the receiving country results in increasing exposure to discrimination [4], and a stronger association between discrimination and poor health status [3]. This may indicate the cumulative effect of discrimination on health due to the country’s racialized structure. For immigrant groups, “becoming American” involves contending with ideologies that render them racial “minorities”, as racialized “others”. “Othering” processes produce and reproduce inequality, with negative consequences on health.

We observed more consistent associations between perceived group discrimination and health outcomes in first generation immigrants living in assimilationist countries. This policy model shares with the inclusive model a civic view of citizenship, facilitating immigrants' naturalization, while, in contrast, confining their cultural and social expression to the private sphere [10]. This contradictory situation in assimilationist countries may explain the broader health gap between those who perceive that they belong to a discriminated group and those who do not.

In this study, we found that perceived discrimination in countries that have an inclusive policy was only associated with depression among men and women and limitation of activity among women. This may be because, while immigrants may feel that they belong to a discriminated group, this may not affect their health negatively because of the effects of active integration and anti-discrimination policies. Additionally, previous studies show that perceived group discrimination is more common among immigrants from former colonies [12], who have a greater presence in some “inclusive” countries, such as the Netherlands or UK.

The sample size available for exclusionist countries was small, which may explain the fact that perceived group discrimination was only significantly associated with a few poor health outcomes in these countries. Moreover, immigrants living in exclusionist countries were mainly from other European countries (approximately half) or Asian countries which may explain the weaker association between perceived discrimination and poor health outcomes. In contrast, African and Latin American immigrants were relatively more common in countries with other integration policy models. Additionally, immigrants in exclusionist countries may be subject to greater and more widespread legal and socioeconomic barriers that affect their health, beyond the mere experience of discrimination [23,24].

### Strengths and Limitations

To our knowledge, this is the first study that analyses perceived discrimination and health outcomes in immigrant groups in Europe, and that comparing first and second generation immigrants and different national immigrant integration policy models. The health implications of immigration policies have received little attention [3], and have not been related to discrimination and health outcomes [1], which highlights the importance of this study.

A limitation of the study is its cross-sectional design, which precludes causal interpretations. However the associations between discrimination and health have also been reproduced with longitudinal design [8]. Another limitation is that our analysis of national integration policies does not account for the heterogeneity between individual countries in each group [23]. For example, Lorant and Bhopal [25] compared policies for tackling ethnic inequalities in health in two countries with an inclusive model, Belgium and Scotland, and found that the latter has provided a far better and more comprehensive response. As has been commented above, a third limitation is the small size of the sample of individuals with an immigrant background, especially second generation immigrants and in exclusionist countries. This small sample size prevented us from exploring associations between perceived discrimination and health according to country of residence, and from teasing out specific groups that could have higher levels of perceived discrimination, such as those from countries where a different race predominates.

## 5. Conclusions and Recommendations

In this study we have found that perceived group discrimination is associated with poor health outcomes in first generation immigrants from low-income countries who live in European countries. These associations are more significant in countries with assimilationist immigration policies. Public policies on integration of immigrant groups are important for reducing discrimination and its related health outcomes.
